# Effects of uric acid-lowering therapy on the progression of chronic kidney disease: a systematic review and meta-analysis

**DOI:** 10.1080/0886022X.2018.1456463

**Published:** 2018-04-05

**Authors:** Xuemei Liu, Tingting Zhai, Ruixia Ma, Congjuan Luo, Huifang Wang, Liqiu Liu

**Affiliations:** Department of Nephrology, The Affiliated Hospital of Qingdao University, Qingdao, China

**Keywords:** Uric acid, chronic kidney disease, uric acid-lowering therapy, randomized controlled trials

## Abstract

**Objectives:** Whether uric acid levels were associated with the progression of chronic kidney disease (CKD) remained controversial. This meta-analysis was aimed to assess the effect of lowering serum uric acid therapy on the progression of CKD to clarify the role of uric acid in the progression of CKD indirectly.

**Methods:** Pubmed, Embase, the Cochrane library, CBM were searched for randomized controlled trials (RCTs) that assessed the efficiency of lowering serum uric acid therapy on the progression of CKD without language restriction. Summary estimates of weighted mean differences (WMDs) and relative risk (RR) were obtained by using random-effect or fixed-effect models. Sensitivity analyses were performed to identify the source of heterogeneity.

**Results:** A total of 12 randomized controlled trials with 832 CKD participants were included in the analysis. Pooled estimate for eGFR was in favor of lowering serum uric acid therapy with a mean difference (MD) of 3.88 ml/min/1.73 m^2^, 95% CI 1.26–6.49 ml/min/1.73 m^2^, *p* = .004 and this was consistent with results for serum creatinine. The risk of worsening of kidney function or ESRD or death was significantly decreased in the treatment group compared to the control group (RR 0.39, 95% CI 0.28–0.52, *p*< .01).

**Conclusions:** Uric acid-lowering therapy may be effective in retarding the progression of CKD. Further randomized controlled trials should be performed to confirm the effect of lowering serum uric acid therapy on the progression of CKD.

## Introduction

Chronic kidney disease (CKD) has become a global public health issue [1]. The prevalence of CKD in the United States in 2012 (not including end-stage renal disease) was estimated at 13.6% by the US Renal Data System [2] and the prevalence of CKD was as high as 10.8% in a survey of a nationally representative sample of Chinese adults [3]. The burden of CKD was not only restricted to progress to end-stage renal disease (ESRD) requiring renal replacement therapy, but also associated with high mortality [4], cardiovascular events [5], hospitalization and healthcare costs [6]. Therefore, early detection and intervention of associated risk factors are essential for delaying, even preventing CKD.

Many risk factors for development and progression of CKD have been recognized, such as hypertension [7], proteinuria [8], diabetes [9]. Hyperuricemia has been considered to be associated with adverse outcomes in CKD due to reduced glomerular filtration rate. Furthermore, recently some studies have proposed that elevated serum uric acid is not merely a consequence, but a novel risk factor in the development and progression of CKD. Several animal experiments have suggested that elevated serum uric acid is a risk factor for development and progression of renal disease. Iseki K [10] in Japan has demonstrated an association between higher serum uric acid levels and greater risk of CKD incidence, whereas some epidemiological studies have reported no significant relationship between hyperuricemia and progression of kidney disease and development of kidney failure [11]. Thus, whether elevated serum uric acid is a risk factor for the progression of CKD has not reached a consensus.

Although chronic kidney disease is not reversible, the progression of CKD can be preventable by appropriate treatment. It is meaningful to examine whether lowering serum uric acid therapy can slow CKD progression. So we performed a meta-analysis about the effect of lowering serum uric acid therapy on the progression of CKD to clarify the role of uric acid in the progression of CKD indirectly.

## Materials and methods

### Search strategy

Literature searches for all articles published until May 2017 were conducted using Pubmed, Embase, the Cochrane library, CBM. The search terms were as follows: ‘chronic kidney disease’, ‘chronic renal insufficiency’, ‘CKD’, ‘allopurinol’, ‘febuxostat’, ‘probenecid’, ‘sulfinpyrazone’, ‘benzbromarone’ and ‘uric acid lowering therapy’ (see supplementary file for search strategy). No language restrictions were applied. If necessary, we corresponded with authors of original articles by e-mail. The search process was performed and confirmed by two investigators.

### Selection criteria

Included studies should fulfill the following inclusion criteria: (1) studies enrolled adult patients with CKD, excluded patients in ESRD. Chronic kidney disease was defined according to the KDOQI guideline, or as defined by study authors, (2) only experimental group was given uric acid lowering therapy, (3) studies were randomized controlled trials, (4) studies reported the following renal outcomes: changes in GFR or serum creatinine, incidence of worsening of renal function or ESRD or death.

Exclusion criteria: (1) studies reporting on patients with ESRD or acute renal impairment or renal carcinoma or patients requiring dialysis or kidney transplant; (2) children; (3) reviews, commentary articles, and editorials.

### Data extraction

Two investigators independently extracted data for included studies using standardized forms. The following data were extracted: author name, publication year, age, sex, sample size, follow-up duration, baseline kidney function, change in kidney function (reported as GFR or serum creatinine concentration or creatinine clearance) from baseline to end of follow-up, progression to worsening of kidney function, incidence of ESRD. Disagreements on extracted data were resolved by discussing between two investigators and consulting others if necessary.

### Quality assessment

The quality of the included studies was assessed according to Cochrane criteria guidelines [12]. The following six items were assessed: (1) random sequence generation; (2) allocation concealment; (3) blinding of participants, personnel, outcome assessment; (4) incomplete outcome data; (5) selective reporting; (6) any other bias (e.g., insufficient rationale, study design). Two researchers evaluated the quality independently. Any disagreement between them was settled by discussing with a third specialist until a consensus was reached.

### Statistical analysis

Statistical analyses and graph formation were performed using Rev man 5.3 software. We used relative risk (RR) with 95% confidence interval (CI) to assess the results of dichotomous outcomes. For continuous outcomes, we calculated the mean differences (MD) with 95% CI. The I^2^ value was carried out to assess heterogeneity across studies, I^2^ value > 50% was considered to indicate relevant heterogeneity. If test for heterogeneity was not significant, summary estimates of RRs, MDs and 95% CIs for the estimates were derived using a fixed effects model; otherwise, a random effects model was used. We also conducted a sensitivity analysis by removing each individual trial from the meta-analysis for the presence of heterogeneity. Sensitivity analyses were performed using Stata 11.0. The standard deviations (SDs) of the change in GFR or serum creatinine concentration or creatinine clearance, when not reported in the studies, were calculated according to the equations provided by the Cochrane Handbook for Systemic Review and Follmann D’s method for overview of clinical trials with continuous variables [13]. The study was performed in compliance with the Preferred Reporting Items for Systematic Reviews and Meta-Analyses (PRISMA) guidelines and the review has not been registered.

## Results

### Study selection and characteristics of included studies

**Figure 1.**
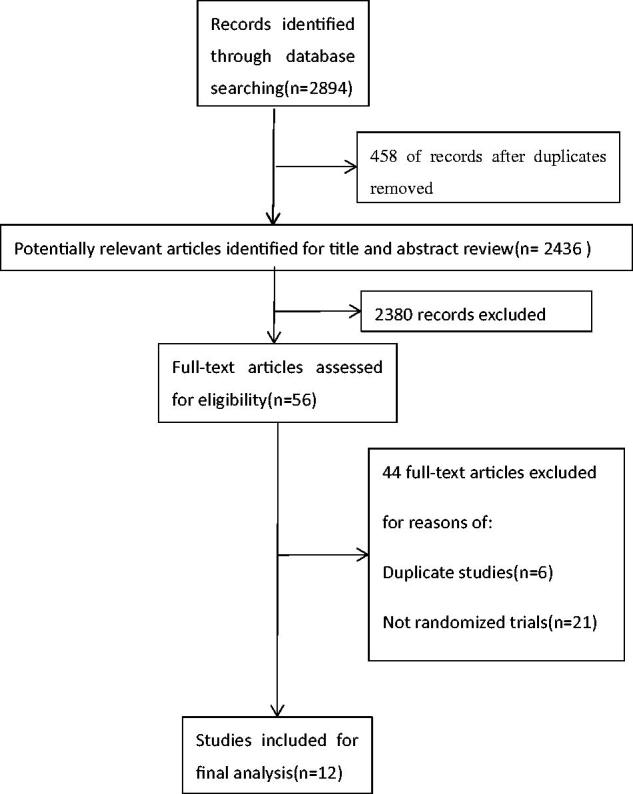
Flow chart of study selection.

We identified 2894 articles from our initial search, of which 458 were excluded by deduplication. We ruled out the inconsistent, nonclinical trials and nontherapeutic studies in the remaining articles. Finally, 12 trials [14–25] involving 832 patients fulfilled the inclusion criteria for the meta-analysis (Figure 1). These studies were randomized controlled trials. The investigated therapies were allopurinol or febuxostat. The follow-up duration ranged from 4 months to 24 months. The subjects were divided into control group and treatment group randomly. The study basic characteristics were shown in Table 1.

**Table 1.** Characteristics of included studies in the meta-analysis.

				Men (%)		Age (years)					
Study	Year of publication	Nation	Sample size	Treatment group	Control group	Treatment group	Control group	Population	Duration of follow-up	Treatment	Control
Deng	2010	China	61	48	56	60.0 ± 11.1	58.8 ± 9.4	Scr level 133∼442umol/L	12 months	allopurinol 100–300 mg/d	No treatment
Goicoechea	2010	Spain	113	Not reported	Not reported	72.1 ± 7.9	71.4 ± 9.5	eGFR lower than 60 ml/min	24 months	allopurinol 100 mg/d	Usual therapy
Kao	2011	UK	53	59	46	70.6 ± 6.9	73.7 ± 5.3	stage 3 CKD and LVH	9 months	allopurinol 300 mg/d	Placebo
Lei	2009	China	57	69	68	48.6 ± 10.2	49.5 ± 9.8	Scr level 133∼442umol/L	12 months	allopurinol 100 to 200 mg/d	No treatment
Liu	2007	China	47	67	57	45.6 ± 12.5	46.5 ± 13.8	Scr level 120∼400umol/L	12 months	allopurinol 100 to 200 mg/d	No treatment
Momeni	2010	Iran	40	45	45	56.3 ± 10.6	59.1 ± 10.6	type 2 diabetes mellitus and diabetic nephropathy (proteinuria, at least 500 mg/24 h and a serum creatinine level less than 3 mg/dL)	4 months	allopurinol 100 mg/d	Placebo
Shen	2010	China	51	69	65	47.1 ± 11.8	47.6 ± 12.4	Scr level 133∼442umol/L	12 months	allopurinol 100 to 200 mg/d	No treatment
Shi	2012	China	40	62	47	39.7 ± 10.0	40.1 ± 10.8	Scr level < 3mg/dl	6 months	allopurinol 100–300 mg/d	Usual therapy
Sircar	2015	India	93	64	77	56.2 ± 10.9	58.4 ± 14.5	eGFR of 15 to 60ml/min/1.73m^2^	6 months	febuxostat 40mg/d	Placebo
Siu	2006	China	51	31	54	47.7 ± 12.9	48.8 ± 16.8	daily proteinuria greater than 0.5 g and/or an elevated serum creatinine (Cr) level greater than 1.35 mg/dL (>120umol/L) at baseline	12 months	allopurinol 100 to 300 mg/d	Usual therapy
Tan	2011	China	140	51	51	59.3 ± 9.2	58.6 ± 8.3	T_2_DM, proteinuria at least 500 mg/24 h, eGFR 30-60 ml/min/1.73 m2	24 months	allopurinol	No treatment
Zhou	2009	China	86	44	41	58.7 ± 8.9	59.3 ± 7.8	daily proteinuria greater than 0.5 g and/or eGFR lower than 60 ml/min (at least 3months)	6 months	allopurinol 100 to 200 mg/d	No treatment

### Quality assessment

The concrete quality assessments of the included studies were shown in Figure 7. Only one trial [19] performed an intention-to-treat analysis for lost follow-up visits. Three trials [16,17,19] were randomized, double-blind and placebo-controlled studies. One trial [18] conducted a open-label study.

### Effect of uric acid-lowering therapy on eGFR

The data were extracted and pooled from five studies. Two trials [15,17] directly reported the change in GFR and the SDs of the change in GFR. The changes in GFR in the other three trials were derived from GFR between baseline and final levels; the SDs of the change in GFR were calculated according to the Cochrane Handbook for Systemic Review and Follmann D’s method [18,19,22].

**Figure 2.**
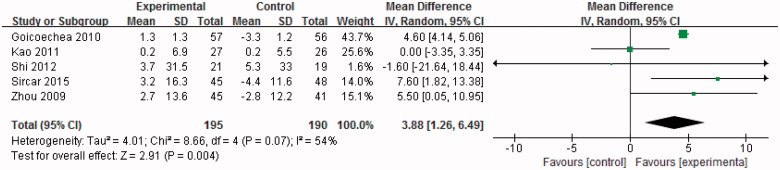
Effect of uric acid-lowering therapy on eGFR.

Compared with the control group, the pooled estimate suggested a significant absolute change in GFR and indicated a significant benefit in patients receiving uric acid lowering therapy (WMD 3.88 mL/min/1.73 m^2^, 95%CI 1.26–6.49, *p* = .004) (Figure 2).

### Effect of uric acid-lowering therapy on SCr

The changes in SCr in these trials were derived from SCr between baseline and final levels; the SDs of the change in SCr were calculated according to the Cochrane Handbook for Systemic Review and Follmann D’s method.

**Figure 3.**
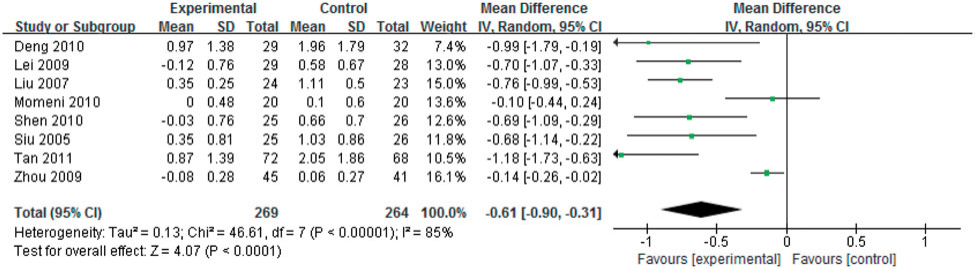
Effect of uric acid-lowering therapy on SCr.

The pooled estimate for the change in SCr between uric acid lowering therapy group and control group was −0.61 mg/dl (95%CI −0.90 to −0.31, *p* < .01) based on data from eight studies, indicating a significant benefit in patients with uric acid-lowering therapy. Significant heterogeneity for this outcome was found (Chi-squared = 46.61, I^2^ = 85%, *p* < .01) (Figure 3).

**Figure 4.**
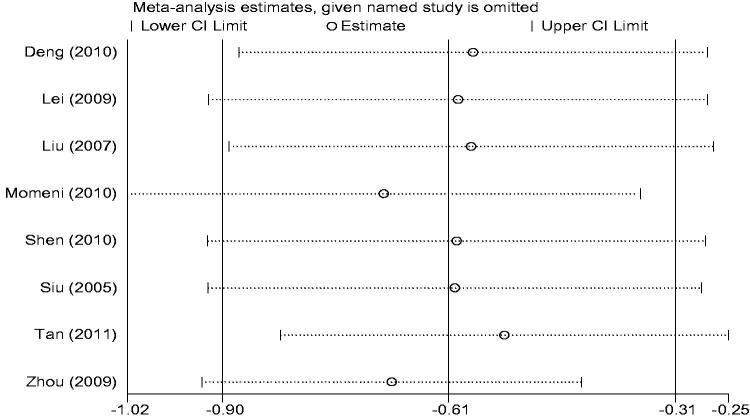
Sensitivity analyses.

**Figure 5.**
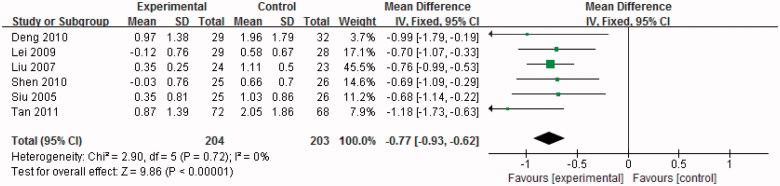
Effect of uric acid-lowering therapy on SCr (excluding the two studies).

The sources of heterogeneity were investigated by sensitivity analyses. Sensitivity analyzes showed that exclusion of any one study from the analysis did not alter the overall findings (Figure 4). Furthermore, excluding the two studies [16,22] that contributed most to the overall estimate also did not materially alter the results (WMD −0.77 mg/dl, 95%CI −0.93 to −0.62, *p* < .01) (Figure 5).

### Effect of uric acid-lowering therapy on renal outcome

We defined study end points as followed: (1) worsening of renal function; (2) end-stage renal disease requiring dialysis therapy; and (3) death.

**Figure 6.**
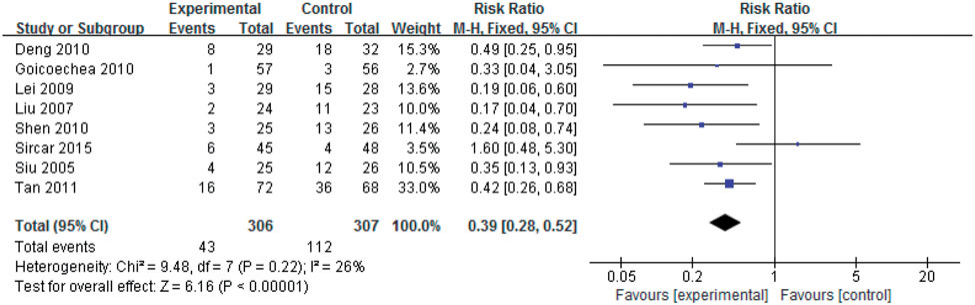
Effect of uric acid-lowering therapy on renal outcome.

Eight trials reported data on renal outcome of uric acid-lowering therapy. Significantly more patients in the control group showed deterioration in kidney function compared with the treatment group (RR 0.39, 95%CI 0.28–0.52, *p* < .01). No heterogeneity was detected among studies (I^2^ = 26%, *p* = .22) (Figure 6).

**Figure 7.**
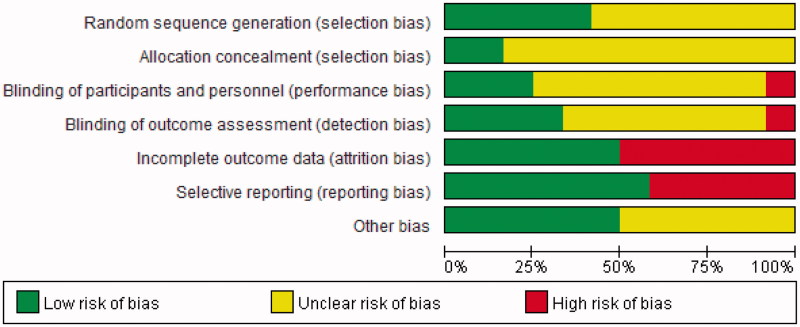
Risk of bias graph.

## Discussion

Hyperuricemia was merely considered as a marker or complication of renal impairment for CKD patients without gout in the past. There are growing studies about factors that promoting progression of CKD and damages that caused by hyperuricemia, and more attention is paid to the effects of hyperuricemia on the progression of CKD. Some studies have suggested that elevated serum uric acid is an independent risk factor for renal disease progression. However, whether uric acid-lowering therapy can retard the progression of CKD is unclear. The related studies retrieved are restricted to small sample clinical studies.

In the present study, we review the role of uric acid-lowering therapy in the progression of CKD. The results of our meta-analysis demonstrated that improved kidney function in CKD patients was associated with uric acid-lowering therapy and early intervention that decreased SUA levels may retard the development of CKD. Though there was significant heterogeneity among eight trials about effect of UALT on SCr, sensitivity analysis was conducted to explain heterogeneity. Momeni and Zhou reported allopurinol therapy lowered serum creatinine concentration in patients with proteinuria. But excluding these two studies found a similar result. Similarly, eight trials reported that the risk of the progression of end-stage renal disease was reduced using uric acid-lowering therapy.

There were a number of mechanisms of the effects of hyperuricemia on the progression of CKD. Traditionally, hyperuricemia resulted in intraluminal crystals in collecting ducts of nephrons, which was similar to the way it causes gout. Uric acid crystals adhere to the surface of renal epithelial cells and induce an inflammatory response [26]. Monocyte chemoattractant protein-1 may be one of the chemokine mediators associated with the localized inflammation [27]. Also, massive uricosuria with intrarenal crystal deposition obstructed renal tubular and increased risk of kidney stone formation to reduce the GFR. On the other hand, more and more studies suggested that uric acid directly stimulated proliferation of vascular smooth muscle cells in the afferent arterioles [28] and caused an increase in glomerular hydrostatic pressure, which induced glomerular hypertension and resulted in glomerular hypertrophy and sclerosis [29]. Also, increasing uric acid levels could induce oxidative stress and endothelial dysfunction, resulting in elevated renal vascular resistance and reduced renal blood flow [30]. In animal study, hyperuricemia rats showed increased kidney inflammation (TNF-α), fibrotic(TGF-β) and oxidative (HO-1) markers, along with pathologically confirmed kidney fibrosis [31,32]. In addition, a recent renal biopsy study of 167 Japanese patients with CKD found that an SUA level above 7.2 mg/dL was associated with renal arterial wall thickening and hyalinosis consistent with renal arteriolopathy after adjusting for age, sex, hypertension, diabetes and eGFR [33]. Therefore, it is possible that uric acid-lowering therapy can slow the progression of CKD theoretically.

The pooled estimates for eGFR and serum creatinine were in favor of lowering serum uric acid therapy in current study. This meta-analysis included reducing urate production with xanthine oxidase inhibitors rather than uricosuric increasing uric acid excretion exactly. Xanthine oxidase inhibitors including allopurinol and febuxostat inhibited oxygen species (ROS) formation and also improved several different measures of endothelial dysfunction in CKD [17,^34^]. Moreover, xanthine oxidase inhibitors may decrease glomerular hydrostatic pressure and alleviate renal damage through reducing uric acid level. It was difficult to interpret whether nephroprotective effect of urate-lowering therapy was caused by lowering uric acid or other effects independent of serum uric acid reduction itself [35]. Our meta-analysis concluded that the risk of worsening of kidney function or ESRD or death was significantly decreased after lowering serum uric acid treatment. As the CKD patients of the current study were receiving ACEIs or ARBs more or less, we assumed that the additional renoprotective effect may be associated with those drugs to improve the renal prognosis of CKD patients.

This systematic review summarized the available evidence on the efficacy of lowering uric acid therapy in slowing CKD progression. To our knowledge, this was the newest systematic review on this topic. Bose et al [36] evaluated the efficacy of allopurinol as a renoprotective agent in a systematic review of 476 participants. There was no significant difference in the change in GFR between the allopurinol and control groups. However, their enrolled patients were not restricted to CKD patients and it was in the context of the general population. Subgroup analysis according to baseline CKD status showed that there was no significant difference in the change in GFR from baseline between the allopurinol and control groups for participants with CKD, but the availability of data from only three studies. In our meta–analysis, we enrolled CKD patients with mild to moderate renal impairment not ESRD and requiring dialysis, who were worth treating mostly, and early intervention may improve the renal prognosis of those patients. Our meta-analysis included CKD patients of five trials proved the change in GFR from baseline was in favor of uric acid-lowering therapy. This result was consistent with data on change in serum creatinine and renal outcome, indicating lowering uric acid may have efficacy for delaying progression of CKD. Kanji et al [37] drew similar conclusions as our systematic review, but they searched up to 2013. Our meta-analysis renewed search date. And this meta-analysis included outcome (1) worsening of renal function; (2) end-stage renal disease requiring dialysis therapy; and (3) death, while there were insufficient data on incidence of ESRD in Kanji’s [37] enrolled studies. Furthermore, febuxostat as a novel drug selectively inhibits xanthine oxidase with apparently stronger hypouricemic effects than allopurinol [38]. It does not require dose adjustment for mild to moderate renal impairment and has renoprotective effect independent of serum uric acid reduction [34]. Randomized controlled trials about the effects of febuxostat on CKD were rare, but one trial in our meta-analysis about febuxostat was used.

Despite its strengths, this systematic review had several limitations. First, we did not have individual data to calculate the standard deviations of the change in SCr before and after receiving uric acid-lowering therapy when not reported in the studies. Second, we did not have sufficient individual data to assess the impact of traditional risk factors, such as hypertension, on the efficiency of uric acid-lowering therapy on retarding the deterioration of kidney function. Thirdly, due to the restriction of amount of enrolled trials, we cannot compare allopurinol with febuxostat on the efficiency of lowering uric acid.

In addition, the clinical studies included in this paper and the other system reviews ignored an important issue – a fundamental component of gout – changes in the miscible pool of urate. The results of Benedict’s study [39] indicated that there were some 1200 mg of miscible uric acid normally, but in the gouty patients, the magnitude of this miscible pool exceeded the normal levels by as much as 15-fold. So large was the quantity of promptly miscible uric acid as to indicate that at least a portion of this material must reside in the solid phase rather than in solution in body fluids. Thus, miscible uric acid in the body may be divided into the following three compartments: (1) In solution in plasma water; (2) In solution in nonplasma body water; (3) In the solid phase. Scott’s [40] research suggested that there was a significant association between the level of serum uric acid and the exchangeable pool size in the range of values, but this may not be valid in patients with very high pool sizes and perhaps where there was acute elevation of the serum uric acid. In other words, the serum urate level was likely not an accurate surrogate marker of the size of the miscible pool in gouty patients. Therefore, we suggest that in CKD patients with gout, the restoration of the miscible pool should be one of the indicators of concern in future research. Only when the miscible pool size returns to normal can it be fully evaluated the effect of uric acid-lowering treatment on the progression of renal failure.

## Conclusions

Lowering uric acid therapy may play an important role in delaying the progression of CKD. Although at present there is no guideline to recommend the routine use of uric acid-lowering drugs to slow the progression of CKD, but more trials have justified the necessity and benefits of lowering serum uric acid level. Furthermore, more double-blind, randomized, placebo-controlled, multicenter studies with high quality were required to assess the impact of uric acid-lowering therapy on CKD progression.

## Supplementary Material

Search Strategies
