# Supplementary material for: Effects of uric acid-lowering therapy on the progression of chronic kidney disease: a systematic review and meta-analysis
Source: Ren Fail. 2018 Apr 5;40(1):289–97. doi: 10.1080/0886022X.2018.1456463 (PMC6014338; doi:10.1080/0886022X.2018.1456463)
Supplement: Search Strategies [file IRNF_A_1456463_SM9061.docx]

**Supplemental data: Search Strategies**

Ovid MEDLINE(R) and Pubmed

1. chronic kidney disease.tw.
2. chronic kidney insufficiency.tw.
3. chronic kidney failure.tw.
4. chronic renal insufficiency.tw.
5. chronic renal failure.tw.
6. nephropathy.tw.
7. exp chronic kidney disease/
8. exp chronic kidney insufficiency/
9. exp chronic kidney failure/
10. exp chronic renal insufficiency/
11. exp chronic renal failure/
12. 1 or 2 or 3 or 4 or 5 or 6 or 7 or 8 or 9 or 10 or 11
13. allopurinol.tw.
14. febuxostat.tw.
15. probenecid.tw.
16. sulfinpyrazone.tw.
17. Benzbromarone.tw.
18. xanthine oxidase inhibitor.tw.
19. uric acid lowering therapy.tw.
20. urate lowering therapy.tw.
21. gout suppressant.tw.
22. exp allopurinol/
23. exp febuxostat/
24. exp probenecid/
25. exp sulfinpyrazone/
26. exp benzbromarone/
27. exp xanthine oxidase/
28. exp gout suppressant/
29. 13 or 14 or 15 or 16 or 17 or 18 or 19 or 20 or 21 or 22 or 23 or 24 or 25 or 26 or 27 or 28

30.12 and 29

Cochrane Library

1. allopurinol:ti,ab,kw
2. febuxostat:ti,ab,kw
3. probenecid:ti,ab,kw
4. sulfinpyrazone:ti,ab,kw
5. benzbromarone:ti,ab,kw
6. xanthine oxidase inhibitor:ti,ab,kw

窗体底端

1. uric acid lowering therapy:ti,ab,kw
2. urate lowering therapy:ti,ab,kw
3. gout suppressant:ti,ab,kw
4. 1 or 2 or 3 or 4 or 5 or 6 or 7 or 8 or 9
5. chronic kidney disease:ti,ab,kw
6. chronic kidney failure:ti,ab,kw
7. chronic kidney insufficiency:ti,ab,kw
8. chronic renal failure:ti,ab,kw
9. chronic renal insufficiency:ti,ab,kw
10. nephropathy:ti,ab,kw

窗体底端

1. 11 or 12 or 13 or 14 or 15 or 16
2. 10 and 17

CBM

1. (("chronic kidney disease") OR "chronic kidney insufficiency") OR "chronic renal failure"

2(((("allopurinol" OR "febuxostat") OR "benzbromarone") OR "probenecid") OR "uric acid lowering therapy"

3. 1 and 2
